# Acquired resistance to irradiation or docetaxel is not associated with cross-resistance to cisplatin in prostate cancer cell lines

**DOI:** 10.1007/s00432-022-03914-5

**Published:** 2022-01-12

**Authors:** Lukas Donix, Holger H. H. Erb, Claudia Peitzsch, Anna Dubrovska, Manuel Pfeifer, Christian Thomas, Susanne Fuessel, Kati Erdmann

**Affiliations:** 1 Department of Urology, Faculty of Medicine, Technische Universität Dresden, Fetscherstr. 74, 01307 Dresden, Germany; 2 National Center for Tumor Diseases (NCT), Dresden, Germany: German Cancer Research Center (DKFZ), Heidelberg, Germany; Faculty of Medicine and University Hospital Carl Gustav Carus, Technische Universität Dresden, Dresden, Germany; Helmholtz-Zentrum Dresden-Rossendorf (HZDR), Dresden, Germany; 3 National Center for Radiation Research in Oncology (OncoRay), Faculty of Medicine, Technische Universität Dresden, Dresden, Germany; 4 Helmholtz-Zentrum Dresden-Rossendorf, Institute of Radiooncology - OncoRay, Dresden, Germany; 5 German Cancer Consortium (DKTK), Partner Site Dresden, Dresden and German Cancer Research Center (DKFZ), Heidelberg, Germany; 6 Institute of Legal Medicine, Faculty of Medicine, Technische Universität Dresden, Dresden, Germany

**Keywords:** Cisplatin, Docetaxel, mCRPC, NEPC, Prostate cancer, Radiotherapy

## Abstract

**Purpose:**

Platinum chemotherapy can be considered to treat metastatic castration-resistant prostate cancer (mCRPC) with features of neuroendocrine differentiation. However, platinum compounds are generally only applied after the failure of multiple prior-line treatment options. This study investigated whether acquired resistance against ionizing radiation or docetaxel chemotherapy—two commonly applied treatment modalities in prostate cancer—influences the cisplatin (CDDP) tolerance in mCRPC cell line models.

**Methods:**

Age-matched parental as well as radio- or docetaxel-resistant DU145 and PC-3 cell lines were treated with CDDP and their sensitivity was assessed by measurements of growth rates, viability, apoptosis, metabolic activity and colony formation ability.

**Results:**

The data suggest that docetaxel resistance does not influence CDDP tolerance in all tested docetaxel-resistant cell lines. Radio-resistance was associated with sensitization to CDDP in PC-3, but not in DU145 cells. In general, DU145 cells tolerated higher CDDP concentrations than PC-3 cells regardless of acquired resistances. Furthermore, non-age-matched treatment-naïve PC-3 cells exhibited significantly different CDDP tolerances.

**Conclusion:**

Like patients, different mCRPC cell lines exhibit significant variability regarding CDDP tolerance. The presented in vitro data suggest that previous radiation treatment may be associated with a moderate sensitization to CDDP in an isogenic and age-matched setting. Therefore, previous radiotherapy or docetaxel chemotherapy might be no contraindication against initiation of platinum chemotherapy in selected mCRPC patients.

**Supplementary Information:**

The online version contains supplementary material available at 10.1007/s00432-022-03914-5.

## Introduction

In men, prostate cancer (PCa) has the second-highest incidence among all cancers in the developed world and was responsible for 6.8% of cancer-related mortality worldwide in 2020 (Sung et al. 2021). Furthermore, the Global Cancer Observatory projects worldwide PCa cases to increase by 60% in the next two decades driven by population growth and rising life expectancy (WHO 2021). For locally confined PCa, radical prostatectomy and external beam radiotherapy are the treatment options with curative intent. While radical prostatectomy is generally the more prevalent method, the average utilization of radiotherapy ranges from 11.8% in Germany to 38.4% in the US (Hager et al. 2015). Metastatic castration-sensitive PCa can be treated by androgen deprivation combined with docetaxel (DTX) chemotherapy, the CYP17 inhibitor abiraterone or newer generation antiandrogens (Cornford et al. 2021). Depending on the prior treatment regimen and genetic features, androgen-signaling modulators such as abiraterone and antiandrogens, taxane-based chemotherapy with DTX or cabazitaxel as well as poly(ADP ribose)polymerase (PARP) inhibitors constitute the current clinical standard of care for metastatic castration-resistant PCa (mCRPC) (Cornford et al. 2021). Consecutive therapy modalities after progression on taxanes, androgen-signaling-targeted therapies and PARP inhibitors are scarce and hardly standardized.

Neuroendocrine (NE) prostate cancer (NEPC) is a variation of PCa that can be treated with platinum chemotherapy. Therapies consisting of either cisplatin (CDDP) or carboplatin in combination with taxanes or etoposide achieve a median overall survival of usually less than a year in this terminal disease (Aparicio et al. 2013; Corn et al. 2019; Culine et al. 2007; Flechon et al. 2011; Papandreou et al. 2002; Steineck et al. 2002). NEPC is characterized by a diminished response to androgen ablation and commonly exhibits characteristics of small cell histology (predominant or partial) and expression of NE markers such as neuron-specific enolase (NSE), chromogranin A (CGA) and synaptophysin (SYP) (Aparicio et al. 2013; Corn et al. 2019; Culine et al. 2007; Flechon et al. 2011; Papandreou et al. 2002; Steineck et al. 2002). While NEPCs are rare at primary PCa diagnosis (< 1% of cases), prospective biopsies of mCRPC tissue revealed NE features in 11.2% of the samples (Abida et al. 2019) and autopsies revealed their presence in 10–20% of men dying with mCRPC (Shah et al. 2004; Tanaka et al. 2001; Turbat-Herrera et al. 1988).

Besides NEPC, other applications of platinum chemotherapy may emerge in the future. Several groups have published case reports and retrospective studies describing promising responses to platinum chemotherapy in mCRPC with homologous recombination repair (HRR) deficiency (Cheng et al. 2016; Pomerantz et al. 2017; Schmid et al. 2020; Zafeiriou et al. 2019). With 10% of mCRPC-derived tumor specimens having mutations in the HRR gene BRCA2 (Abida et al. 2017), HRR deficiency is common in mCRPC. Platinum-based therapies induce intra- and interstrand DNA crosslinks and have repeatedly shown increased efficacy in HRR-deficient cancer entities including mCRPC (Cheng et al. 2016; Pennington et al. 2014). Furthermore, an interventional phase I study (NCT03275857) currently evaluates CDDP for enzalutamide-refractory mCRPC (NIH 2021). However, as of now, the application of platinum chemotherapy is primarily limited to NEPC treatment.

Refined therapy regimens with a growing variety of efficient androgen-signaling modulators prolongate survival in mCRPC patients and thus, NEPC may become a common late-stage challenge. Additionally, novel applications for platinum chemotherapy besides NEPC may emerge and in summary, the importance of platinum-based therapies for mCRPC management might grow. Because the subset of mCRPC patients initiated on platinum chemotherapy is small and intertumoral genetic diversity overlaps with considerable variety of prior treatment histories, it is challenging for retrospective studies to dissect the influence of prior treatment histories on platinum efficacy.

To address this problem, we investigated putative effects of prior treatment with either ionizing radiation (IR) or DTX on CDDP tolerance in DU145 and PC-3 cells. DU145 and PC-3 are two widely used models for mCRPC. PC-3 cells express the NE markers NSE (van Bokhoven et al. 2003) and CGA (Tai et al. 2011). When xenografted into mice, PC-3 cells exhibit a small cell histology that can be described as reminiscent of NEPC (Tai et al. 2011). DU145 cells, on the other hand, lack NE features (van Bokhoven et al. 2003). Radio-resistant (RR) (Cojoc et al. 2015; Peitzsch et al. 2016) and DTX-resistant (DTXR) (Puhr et al. 2012) DU145 and PC-3 cells as well as their isogenic and age-matched parental cell lines were treated with CDDP in vitro. Their CDDP sensitivity was assessed by measurements of growth rates, viability, apoptosis, metabolic activity and colony formation ability.

## Materials and methods

### Cell lines and culture conditions

Dr. Martin Puhr and Prof. Dr. Zoran Culig provided DU145^Δ^ DTXR, PC 3^Δ^ DTXR and their parental cell lines (Puhr et al. 2012) and Dr. Claudia Peitzsch and Prof. Dr. Anna Dubrovska provided DU145^#^ RR, PC-3^#^ RR and their parental cell lines (Peitzsch et al. 2016). Cell lines are listed in Table 1. The cells were cultured under standard conditions (37 °C, humidified atmosphere with 5% CO_2_) in RPMI-1640 medium (Thermo Fisher Scientific, Waltham, MA, USA) supplemented with 10% fetal bovine serum (Thermo Fisher Scientific). DTXR cells continuously received 10 nM DTX (Merck Millipore, Darmstadt, Germany) to maintain the selection pressure. Cryo-conserved RR cell lines were previously shown to maintain their RR properties for three months without additional conditioning (Peitzsch et al. 2016). Accordingly, RR cells were used for a maximum of six weeks in this study. All cell lines were authenticated by Short Tandem Repeat (STR) analysis (Table S1). For STR analysis, cell line DNA was amplified using PowerPlex 18D System (Promega, Mannheim, Germany) according to manufacturer’s instructions. Capillary electrophoresis was then performed with ABI 3500 Genetic Analyzer (Thermo Fisher Scientific) and analyzed by GeneMapper ID-X software version 1.4 (Thermo Fisher Scientific). All cells were regularly tested for mycoplasma using the Mycoalert™ Mycoplasma Detection kit (Lonza, Basel, Switzerland) according to the manufacturer's instructions.

**Table 1:** Cell lines used in this study

Parental control cell lines	Treatment-resistant sublines	Resistance established by
DU145^Δ^ CTRL & PC-3^Δ^ CTRL	DU145^Δ^ DTXR & PC-3^Δ^ DTXR	Dose escalation^a^ (Puhr et al. 2012)
DU145^#^ CTRL & PC-3^#^ CTRL	DU145^#^ RR & PC-3^#^ RR	Fractionated irradiation^b^ (Cojoc et al. 2015)

^a^DTX tolerance of cells is shown in Fig. S1

^b^Radio-resistance of cells was shown in the cited publication

### Dose–response to chemotherapy

Tolerances for CDDP and DTX were determined by treatment with serial dilutions followed by various readouts. In 96-well plates, 800 DU145 or 2,000 PC-3 cells were seeded per well. For DTX treatment, the cells were cultured to ~ 50% confluence and then treated with 0.1 nM–10 µM DTX for 24 h. Next, the treatment solution was removed, fresh cell culture medium was added and 24 h later metabolic activity and viability were measured by WST-1 assay (Roche, Mannheim, Germany) according to the manufacturer's instructions. WST-1 is metabolized to formazan by the mitochondrial succinate-tetrazolium-reductase system. Thus, the readout is positively proportional to the degree of energy generation via oxidative phosphorylation in metabolically active cells. The formazan absorption was measured at 450 nm with a reference measurement at 620 nm using a Berthold Mithras LB940 microplate reader (Berthold Technologies, Bad Wildheim, Germany).

For CDDP, the cells were treated with 0.01–1.0 µg/mL CDDP 24 h after seeding. Cell growth in CDDP presence was studied by measurement of cell confluence in 6 h-intervals over the course of one week using the IncuCyte S3 Live-Cell Analysis System (Sartorius, Göttingen, Germany). The confluence measurement at 144 h (6 days after seeding) was used to calculate the inhibition curves. Subsequently, the WST-1 assay was performed. Next, adherent cells were fixed with methanol and stained with an aqueous solution of 0.1% crystal violet (Merck Millipore) for 10 min. After thorough washing with water, stained adherent cells were dissolved by adding 100 µL of 0.1 M sodium citrate in 50% ethanol per well and incubating for 30 min on a shaker. Crystal violet absorption was measured at 590 nm using a Berthold Mithras LB940 microplate reader (Berthold Technologies).

### Apoptosis and colony forming assays

Cells were seeded in 6-well plates (15,000 per well for all DU145 lines, 50,000 for PC-3 RR and 30,000 for all other PC-3 lines), cultured to ~ 50% confluence and then treated with 0.01–1.0 µg/mL CDDP or 5.6–560 nM DTX for 24 h. The treatment was removed and 24 h later, non-adherent and adherent cells were harvested with an aqueous solution containing 0.05% trypsin/EDTA (Thermo Fisher Scientific). The cell suspensions were centrifuged, the pellets resuspended in ice-cold PBS and thereafter kept on ice. Viable cells were counted using the Muse^®^ Count & Viability kit on a Muse^®^ Cell Analyzer flow cytometer (Luminex Corporation, Austin, TX, USA) according to the manufacturer's instructions. For measurement of apoptosis based on pan-caspase activity, 4 × 10^4^ viable cells were analyzed using the Muse^®^ Multi Caspase kit (Luminex Corporation) on the Muse^®^ Cell Analyzer according to the manufacturer’s instructions. For colony formation assays, technical triplicates of 100 (for all DU145 cells) or 200 (for all PC-3 cells) viable cells per well were seeded into 6-well plates. At day 8–12 of culture, bright field images of non-stained colonies were taken with the IncuCyte S3 Live-Cell Analysis System and the colonies were counted from these images. The field of view covered 80% of the total surface of the culture wells. Only colonies with > 20 cells were considered. Colonies were then fixed with methanol, stained with an aqueous solution of 0.1% crystal violet and recounted to validate the image-based counting method.

### Statistics

Curve-fitting, calculation of half-maximal inhibitory concentrations (IC_50_), statistical tests and plotting were performed using GraphPad Prism 9 (GraphPad Software, San Diego, CA, USA). For the cell growth tracked by live-cell imaging, non-linear ‘log(agonist) vs. response’ regression with variable slope was used for curve-fitting. The lower plateau was constrained to be ≥ 0 and the upper plateau was constrained to be ≤ 100. When evaluating the response to chemotherapy treatment based on cell confluence at 144 h, on the WST-1 assay or the crystal violet assay, the data were first normalized to the untreated control and then log-transformed. Non-linear ‘log(inhibitor) vs. response’ regression with variable slope was used for curve-fitting and to calculate the IC_50_ values. Due to data normalization, the upper plateaus of the inhibition curves were constrained to equal 1. The lower plateaus were constrained to be ≥ 0.

For colony formation assays, the numbers of colonies in treated groups were normalized to the number of colonies in the untreated group and *x*-fold numbers of cells were plotted. The colony count reduction in response to treatment was modeled by fitting negative exponential growth curves onto the data. The concentration *β* at which colony formation was reduced by 50% was inferred from the curve and is noted as *β*_1/2_ on graphs. Due to previous data normalization, the curves were constrained to cross the *y*-axis at *y* = 1.

All experiments conducted in this study were performed with at least three (up to six) biological replicates and data are depicted as mean ± standard deviation (SD) if not otherwise stated. All statistical tests were done without the assumption of equal variance. Non-paired *t*-tests with Welch’s correction were performed to test for the statistical significance of differences between two groups. For comparisons between multiple groups, Brown-Forsythe and Welch ANOVA combined with Tukey’s test for multiple comparisons were used. *P* values < 0.05 were considered statistically significant.

## Results

### Validation of cell line authenticity and treatment resistance

The identity of all cell lines was confirmed by STR analysis. The observed repeat-length of tetra- and pentanucleotide repeat markers (Urquhart et al. 1994) included in the authentication panel largely matched the expected repeat-length for these markers. DU145 cells exhibited minor deviations of observed versus expected repeat-length more often than PC-3 cells (Table S1). For example, DU145^Δ^ CTRL and DU145^Δ^ DTXR exhibited minor deviations from the STR profile published by ATCC in 7/15 and 11/15 markers, respectively. The instability of STR markers observed in DU145 cell lines may be attributed to the documented DNA mismatch repair (MMR) deficiency in these cells (Boyer et al. 1995; Chen et al. 2001; Lu et al. 2013).

Radio-resistance in DU145^#^ RR and PC-3^#^ RR was previously demonstrated (Peitzsch et al. 2016). To confirm DTX resistance of DTXR cell lines, the cells were treated with serial dilutions of DTX. DTXR cells exhibited a significantly increased DTX tolerance based on the WST-1 assay (IC_50_ of 5.9 nM in DU145^Δ^ CTRL vs 388 nM in DU145^Δ^ DTXR and 8.2 nM in PC-3^Δ^ CTRL vs 305 nM in PC-3^Δ^ DTXR) (Fig. S1a). Colony formation assays (Fig. S1b) confirmed these results.

### CDDP treatment of radio-resistant cells

**Fig. 1:**
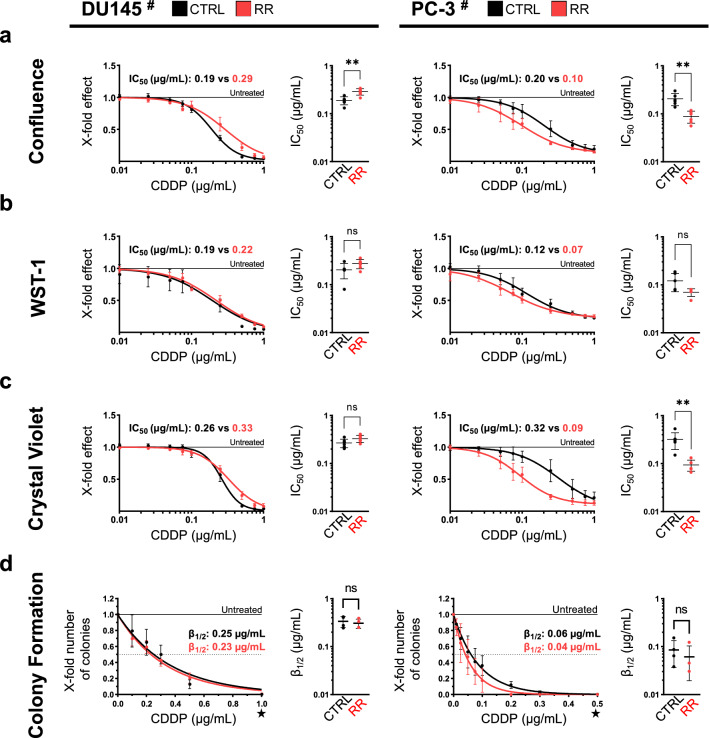
Evaluation of CDDP tolerance in radio-resistant DU145 and PC-3 cells. Radio-resistant cells and their parental controls were treated with CDDP. **a** Cell confluence at 144 h: The *x*-fold confluence of treated cells normalized to untreated cells at 144 h after seeding is plotted. **b** WST-1 assay: The *x*-fold metabolic activity of treated cells normalized to untreated cells is plotted. **c** Crystal violet assay: The *x*-fold viability of treated cells normalized to untreated cells is plotted. **a**–**c** IC_50_ values were calculated from the resulting inhibition curves and are noted in the graphs, whereas IC_50_ values calculated from individual experiments are depicted in the adjacent graphs. **d** Colony formation assay: The *x*-fold number of colonies of treated cells normalized to untreated cells is plotted. The concentrations *β*_1/2_, at which colony formation abilities were reduced by 50%, were inferred from the fitted curves and are noted in the graphs. The ★ symbol highlights the different *x*-axis scaling for DU145^#^ and PC-3^#^ cells. All data are presented as mean ± SD and experiments were repeated for *N* ≥ 3 times. Non-paired *t*-tests with Welch’s correction were performed to test for statistical significance of the differences between RR cells and parental controls or between treated and untreated cells. *ns* not significant, *P < 0.05, **P < 0.01

DU145^#^ RR and PC-3^#^ RR cell lines as well as their non-resistant parental controls were treated with a serial dilution of CDDP and subjected to functional analysis. Cell confluence was tracked over one week in the IncuCyte S3 Live-Cell Analysis System (Fig. S2a). Cell growth under CDDP pressure was comparable between CTRL and RR cells at concentrations lower than 0.1 µg/mL CDDP in DU145 and at concentrations lower than 0.05 µg/mL CDDP in PC-3 cell lines (Fig. S2a). Differences emerged at higher concentrations. DU145^#^ RR cells grew at 0.5 µg/mL CDDP, while the corresponding treatment-naïve DU145^#^ CTRL cells did not proliferate at this concentration. In contrast, PC-3^#^ CTRL cells tolerated CDDP concentrations from 0.1 to 0.25 µg/mL better than PC-3^#^ RR cells (Fig. S2a). Inhibition curves calculated from the cell confluence at 144 h (Fig. 1a) suggest that DU145^#^ RR cells are more resistant and PC-3^#^ RR are more sensitive to CDDP than their corresponding parental control cell lines.

Consecutive to cell growth tracking, cellular viability was measured by WST-1 assay (Fig. 1b). IC_50_ values calculated from the resulting inhibition curves equaled 0.19, 0.22, 0.12 and 0.07 µg/mL CDDP in DU145^#^ CTRL, DU145^#^ RR, PC-3^#^ CTRL and PC-3^#^ RR cells, respectively. Based on the WST-1 assay, no significant differences in CDDP tolerance were observed between RR cells and their parental controls (Fig. 1b).

Subsequently, crystal violet assays were performed (Fig. 1c) determining the total adherent (viable) cell mass. Crystal violet assays revealed no difference between DU145^#^ CTRL and DU145^#^ RR cells. However, the results implied PC-3^#^ RR to be more sensitive to CDDP than PC-3^#^ CTRL (Fig. 1c).

DU145^#^ CTRL and DU145^#^ RR exhibited nearly identical colony formation efficiency after CDDP treatment (Fig. 1d). Colony formation efficiency was slightly higher in PC-3^#^ CTRL vs PC-3^#^ RR cells (Fig. 1d, *P* < 0.05 at 0.1 and 0.2 µg/mL CDDP), which is in line with results obtained from other readouts as described above.

CDDP treatment led to an induction of apoptosis only in DU145^#^ CTRL cells (Fig. S2b). Induction of apoptosis by CDDP was not observed in DU145^#^ RR, PC-3^#^ CTRL or PC-3^#^ RR. However, untreated RR cell lines had a significantly higher basal pan-caspase activity than their parental controls (Fig. S2b).

### CDDP treatment of docetaxel-resistant cells

**Fig. 2:**
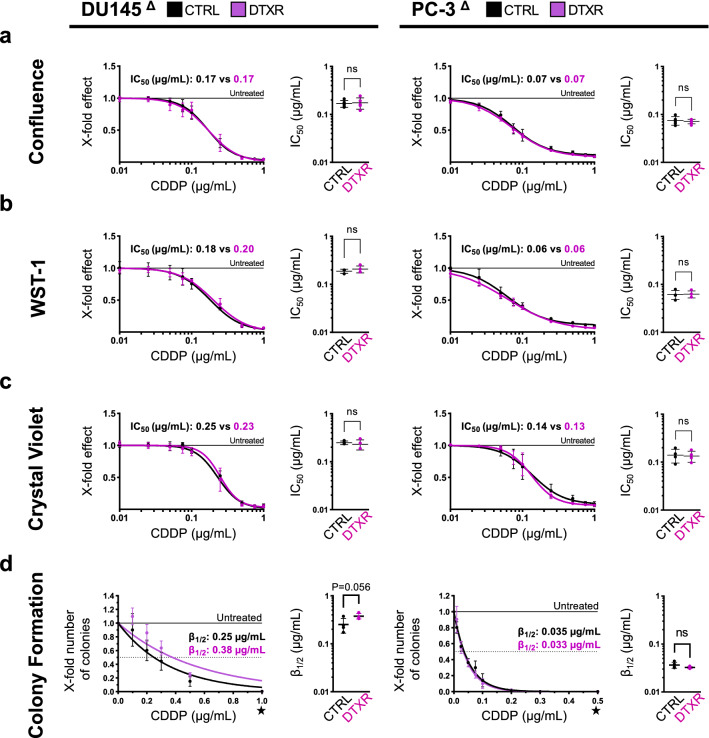
Evaluation of CDDP tolerance in docetaxel-resistant DU145 and PC-3 cells. Docetaxel-resistant cells and their parental controls were treated with CDDP. **a** Cell confluence at 144 h: The *x*-fold confluence of treated cells normalized to untreated cells at 144 h after seeding is plotted. **b** WST-1 assay: The *x*-fold metabolic activity of treated cells normalized to untreated cells is plotted. **c** Crystal violet assay: The *x*-fold viability of treated cells normalized to untreated cells is plotted. **a**–**c** IC_50_ values were calculated from the resulting inhibition curves and are noted in the graphs, whereas IC_50_ values calculated from individual experiments are depicted in the adjacent graphs. **d** Colony formation assay: The *x*-fold number of colonies of treated cells normalized to untreated cells is plotted. The concentrations *β*_1/2_, at which colony formation abilities were reduced by 50%, were inferred from the fitted curves and are noted in the graphs. The ★ symbol highlights the different *x*-axis scaling for DU145^Δ^ and PC-3^Δ^ cells. All data are presented as mean ± SD and experiments were repeated for *N* ≥ 3 times. Non-paired *t*-tests with Welch’s correction were performed to test for statistical significance of the differences between DTXR cells and parental controls or between treated and untreated cells. *ns* not significant, *P < 0.05, **P < 0.01

Analogous to RR cells, DTXR cells and their parental controls were treated with CDDP and growth was studied by continuous imaging. Comparing DTXR cell lines to their age-matched parental controls, cell growth was identical along the entire CDDP serial dilution (Fig. S2c). DU145^Δ^ CTRL and DU145^Δ^ DTXR cells grew in the presence of up to 0.25 µg/mL CDDP, whereas PC-3^Δ^ CTRL and PC-3^Δ^ DTXR cells proliferated in the presence of up to 0.1 µg/mL CDDP (Fig. S2c). IC_50_ values calculated from the inhibition curves based on the cell confluence at 144 h (Fig. 2a) suggested no differences between DTXR cells and their corresponding parental control cell lines.

IC_50_ values based on the WST-1 assay (Fig. 2b) were 0.18, 0.20, 0.06 and 0.06 µg/mL CDDP in DU145^Δ^ CTRL, DU145^Δ^ DTXR, PC-3^Δ^ CTRL and PC-3^Δ^ DTXR cells, respectively. Based on the WST-1 assay, no significant differences in CDDP tolerance were observed between DTXR cells and their parental controls (Fig. 2b). Likewise, consecutive crystal violet assays exhibited no difference between DTXR cells and their corresponding parental control cell lines (Fig. 2c).

DU145^Δ^ DTXR cells exhibited a slightly higher colony formation efficiency compared to DU145^Δ^ CTRL cells at low concentrations of CDDP between 0.1 and 0.3 µg/mL (Fig. 2d, *P* < 0.05 at 0.2 µg/mL CDDP). In PC-3^Δ^ CTRL and PC-3^Δ^ DTXR cells, CDDP inhibited colony formation efficiency to identical degrees along the entire range of tested concentrations (Fig. 2d).

Treatment with CDDP induced pan-caspase activity in all cell lines to comparable degrees, but revealed no difference between DTXR and non-resistant cells (Fig. S2d).

### CDDP tolerance among treatment-naïve control cells

**Fig. 3:**
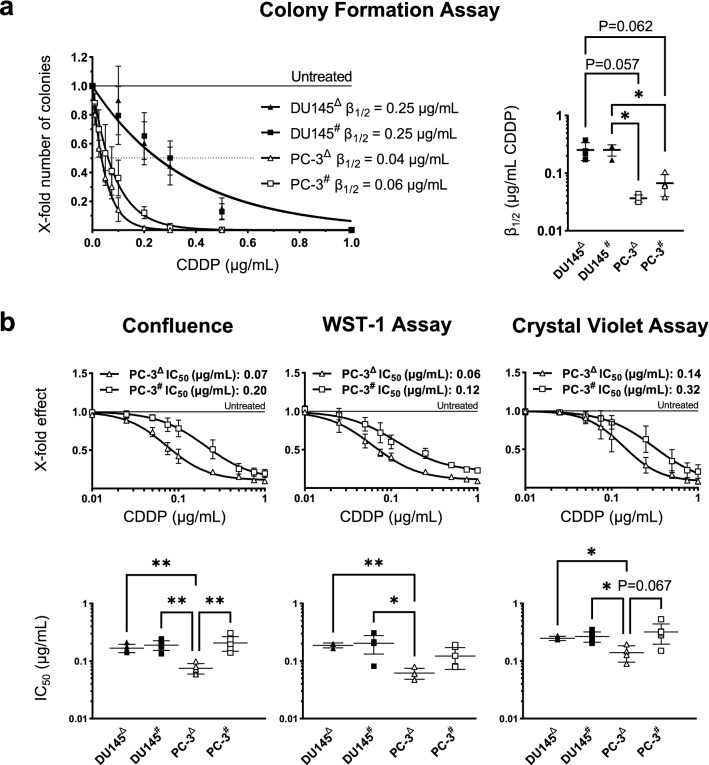
Comparison of CDDP tolerance in control cells. For this figure, the data that were shown in Figs. 1 and  2 were reorganized to allow for an easier comparison of CDDP tolerance between non-isogenic (PC-3 vs DU145) and non-age-matched (PC-3^#^ CTRL vs PC-3^Δ^ CTRL) treatment-naïve control cells. **a** Results of the colony formation assays in all control cell lines are depicted. Adjacent, *β*_1/2_ values calculated from individual experiments (*N* ≥ 3) and the results of an ANOVA with multiple comparisons are shown. **b** Dose–response curves of PC-3^Δ^ CTRL and PC-3^#^ CTRL cells based on the cell confluence at 144 h, the WST-1 assay and the crystal violet assay are shown. DU145^#^ CTRL and DU145^Δ^ CTRL are omitted to allow for a better visual comparison of PC-3^Δ^ CTRL and PC-3^#^ CTRL cells. Beneath, IC_50_ values calculated from individual experiments and the results of an ANOVA with multiple comparisons including DU145^Δ^ CTRL and DU145^#^ CTRL cells are shown. Significant test results (*P* < 0.05) are annotated with asterisks and near significant test results (*P* < 0.1) are annotated with their *P* values. All data are presented as mean ± SD and experiments were repeated for *N* ≥ 3 times. *ns* not significant, *P < 0.05, **P < 0.01

Based on colony formation assays, DU145 cells tolerated higher concentrations of CDDP than PC-3 cells. In contrast to PC-3 cells, DU145 cells maintained residual levels of colony forming ability after treatment with up to 0.5 µg/mL CDDP (Fig. 3a). Based on cell confluence at 144 h, on WST-1 assay and on crystal violet assay the higher tolerance of DU145 CTRL cells was observed only vs PC-3^Δ^ CTRL, but not vs PC-3^#^ CTRL cells (Fig. 3b, bottom row). CDDP tolerance in DU145^Δ^ CTRL compared to DU145^#^ CTRL cells was nearly identical (Fig. 3a, b).

Cell growth under CDDP pressure suggested that PC-3^Δ^ CTRL are more sensitive to CDDP than PC-3^#^ CTRL cells. PC-3^#^ CTRL cells proliferated at up to 0.25 µg/mL CDDP, while in PC-3^Δ^ CTRL growth was abolished at this concentration (compare Fig. S2a, c). Inhibition curves and IC_50_ values based on the cell confluence at 144 h and crystal violet assays also indicated higher CDDP sensitivity of PC-3^Δ^ CTRL than PC-3^#^ CTRL cells (Fig. 3b).

## Discussion

Platinum compounds are generally only applied after the failure of multiple prior-line treatment options. Therefore, we investigated whether acquired resistance against IR or DTX, which are two commonly applied treatment modalities in PCa, influences CDDP tolerance in the mCRPC cell line models PC-3 and DU145. In current clinical practice, platinum chemotherapy is primarily used to treat NEPC patients. The PC-3 cell line does exhibit NE features (Tai et al. 2011; van Bokhoven et al. 2003) and can therefore be considered as a model system for a patient that might be selected for platinum chemotherapy. DU145 cells, on the other hand, do not exhibit NE features (van Bokhoven et al. 2003).

The DTXR cells used in this study exhibited strongly increased DTX tolerance, as shown previously (Puhr et al. 2012) and confirmed within this study. Among others, Puhr et al*.* demonstrated that the DTX-resistant phenotype in these cells was associated with epithelial to mesenchymal transition (EMT)-like changes in cell morphology. This notion was supported by gene expression studies that confirmed upregulation of mesenchymal and downregulation of epithelial markers (Hanrahan et al. 2017; Puhr et al. 2012). EMT has been linked to increased CDDP tolerance in various cancer entities (Ashrafizadeh et al. 2020). In accordance, we confirmed the downregulation of the epithelial cell marker E-cadherin and also the differential expression of genes not directly linked to EMT such as ABCB1—one of the well-studied effector proteins of multi-drug and DTX resistance—in both DTXR cell lines (unpublished). Taken together, DTXR cells exhibit numerous differences at the transcriptomic and proteomic levels compared to their age-matched parental control cells. Despite that, our dose–response experiments unanimously showed no sign of difference in CDDP tolerance between DTXR and control cell lines. This suggests that the cellular adaptations associated with DTX resistance, including EMT, do not affect CDDP tolerance in DU145 and PC-3 human metastatic PCa cell lines.

Previous treatment with IR yielded contradicting results for CDDP tolerance in PC-3^#^ CTRL vs PC-3^#^ RR on one hand and DU145^#^ CTRL vs DU145^#^ RR on the other. DU145^#^ CTRL and DU145^#^ RR generally exhibited comparable CDDP tolerance. However, the cell growth under CDDP pressure and cell confluence at 144 h indicated DU145^#^ RR cells to be slightly more tolerant than DU145^#^ CTRL cells towards CDDP. In contrast, PC-3^#^ RR cells were more sensitive to CDDP treatment than PC-3^#^ CTRL cells based on cell growth, cell confluence at 144 h, crystal violet assays and colony formation assays.

RR cells were obtained by IR treatment, which causes large numbers of DNA lesions randomly distributed across the genome, not all of which are repaired with conservation of the original sequence. The consequence of this is that RR and CTRL cell line pairs are not entirely isogenic, which has been shown for DU145^#^ RR (Seifert et al. 2019). Although DNA lesions caused by IR are randomly distributed, it is possible that PC-3^#^ RR acquired mutations rendering them less capable of tolerating CDDP than non-radiated PC-3^#^ CTRL.

IR-induced DNA lesions include single-strand breaks, double-strand breaks (DSBs), DNA inter-strand crosslinks, and single base modifications (Mladenov et al. 2013). DSBs are considered to be the main drivers of IR cytotoxicity and are repaired through either HRR or variants of non-homologous end joining (Mladenov et al. 2013). Reactive oxygen species generated by IR lead to base modifications, of which 8-oxo-guanine (8-oxoG) is particularly common (David et al. 2007). 8-oxoG lesions are primarily repaired through base excision repair (David et al. 2007). However, transcription-coupled repair (Le Page et al. 2000), nucleotide excision repair (Scott et al. 1999) and MMR (Brierley and Martin 2013) have been implicated as backup and auxiliary repair systems.

HRR and nucleotide excision repair are essential for repairing DNA lesions caused by treatment with platinum compounds such as CDDP (Kelland 2007; Pennington et al. 2014). As elaborated, the diverse types of DNA damage caused by IR treatment require activity of the entire range of DNA-repair pathways. IR treatment may therefore increase the activity of e.g. the HRR pathway, which may in turn enhance a cell’s ability to tolerate CDDP.

However, increased activity of the MMR pathway—which may be a consequence of 8-oxoG mismatches caused by IR—would have a well-established negative effect on CDDP tolerance (Aebi et al. 1996; Fink et al. 1996). Mechanistically, the MMR system recognizes platinum-induced DNA lesions but fails to repair them, fostering apoptosis due to stalled DNA repair complexes (Bassett et al. 2002; Fink et al. 1996; Kelland 2007; Sawant et al. 2015). Therefore, when the activity of the MMR pathway is increased, CDDP tolerance is decreased. Conversely, deficiency for MMR pathway members including MSH2, MSH3, MSH6 and MLH1 can increase platinum resistance (Fink et al. 1996; Goodspeed et al. 2019; Sawant et al. 2015). The detailed role of MMR in the repair of IR-induced 8-oxoG lesions is debated. While some studies suggest that the MMR system can recognize and successfully repair 8-oxoG mismatches (Brierley and Martin 2013), others propose a 'futile repair' mechanism similar to what has been described for platinum-induced lesions (Larson et al. 2003). Studies agree, however, that MMR proteins recognize and interact with 8-oxoG mismatches.

Following this line of thought, increased activity of the MMR system in consequence of the IR treatment could provide a possible explanation for the increased sensitivity to CDDP observed in PC-3^#^ RR vs PC-3^#^ CTRL cells. This effect would not materialize in DU145 cells as these cells are MMR-deficient due to a fatal truncating mutation in the MMR gene *MLH1* (Boyer et al. 1995; Chen et al. 2001; Lu et al. 2013). Indeed, unlike PC-3^#^ RR, DU145^#^ RR cells did not exhibit increased sensitivity to CDDP relative to their control cells and even tolerated slightly higher CDDP concentrations based on cell growth and cell confluence at 144 h.

The presented experiments point out additional observations apart from the comparisons of resistant vs age-matched non-resistant cells. In colony formation assays, treatment-naïve DU145 cells tolerated higher CDDP doses than PC-3 cells. MMR deficiency was already discussed above as a possible driver of increased CDDP tolerance in DU145 cells. Another explanation for increased CDDP tolerance in DU145 relative to PC-3 cells could be given by differences in the NE differentiation status between these two cell lines. While DU145 cells are negative for common markers of NE differentiation, PC-3 cells are positive for NSE (van Bokhoven et al. 2003) and CGA (Tai et al. 2011). Additionally, PC-3 xenografts in mice exhibited expression patterns of NSE, CGA, CK-8 and CD44 as well as histomorphologic features akin to NEPC biopsy samples (Tai et al. 2011). In patients, there is solid evidence that NEPC responds better to platinum treatment than mCRPC without NE features (Aparicio et al. 2013; Humeniuk et al. 2018; Papandreou et al. 2002).

Furthermore, we observed a significant difference in CDDP tolerance comparing the PC-3^#^ CTRL (Cojoc et al. 2015) to PC-3^Δ^ CTRL (Puhr et al. 2012) cells. Both cell lines were purchased from ATCC by their original owners and were authenticated before experimentation in this study. All cell lines were cultured in RPMI-1640 medium supplemented with 10% serum and 2 mM l-glutamine in this study. However, in their original laboratories, PC-3^#^ CTRL received DMEM, while PC-3^Δ^ CTRL received RPMI-1640 (with 10% serum and 2 mM l-glutamine). Naturally, PC-3^#^ CTRL and PC-3^Δ^ CTRL cells are not age-matched and in addition to different historical culture conditions, passage number effects might account for the observed experimental results.

In patients, platinum-based chemotherapies are administered to treat NEPC and recent studies have described enhanced efficacy in mCRPC with HRR deficiency (Cheng et al. 2016; Pomerantz et al. 2017; Schmid et al. 2020; Zafeiriou et al. 2019). The PARP inhibitor olaparib has recently been approved to treat HRR-deficient mCRPC. Therapies involving platinum compounds alone or in combination with PARP inhibition may be a new direction to be evaluated prospectively in this genetic subset of mCRPC patients.

Our work aimed to evaluate the influence of the treatment history on CDDP tolerance and indicates that DTX resistance is not associated with altered CDDP tolerance in mCRPC cell line models. Previous radiation treatment might sensitize cells to CDDP as suggested by comparing PC-3^#^ RR with age-matched PC-3^#^ CTRL cells. However, our data indicated significant differences in CDDP tolerance between DU145 and PC-3 cells as well as between PC-3^#^ CTRL and PC-3^Δ^ CTRL cells, putting the observed effect size of the possible radiation-associated sensitization in PC-3^#^ RR cells into perspective.

In conclusion, prior radiation or DTX treatment are likely not contraindications against the application of CDDP and radiation treatment may be associated with CDDP sensitization. However, this effect may often be masked by the influence of the genetic background. Therefore, to confirm or reject the existence and clinical significance of such an effect, convincing prospective clinical studies would require detailed patient data and sufficiently large cohorts.

## Supplementary Information

Below is the link to the electronic supplementary material.

Supplementary file1 (PDF 1466 KB)

## Data Availability

The data presented in this study are available from the corresponding author upon reasonable request.
