# Supplementary material for: Acquired resistance to irradiation or docetaxel is not associated with cross-resistance to cisplatin in prostate cancer cell lines
Source: J Cancer Res Clin Oncol. 2022 Jan 12;148(6):1313–24. doi: 10.1007/s00432-022-03914-5 (PMC9114061; doi:10.1007/s00432-022-03914-5)
Supplement: Supplementary file 1 — Supplementary file1 (PDF 1466 KB) [file 432_2022_3914_MOESM1_ESM.pdf]

Journal of Cancer Research and Clinical Oncology

Acquired resistance to irradiation or docetaxel is not associated with cross-resistance to cisplatin in prostate cancer cell lines

Lukas Donix [1, 2], Holger H.H. Erb [1], Claudia Peitzsch [2, 3, #], Anna Dubrovska [2, 3, 4, 5], Manuel Pfeifer [6], Christian Thomas [1, 2], Susanne Fuessel [1, 3] and Kati Erdmann [1, 2, 5, *]

1 Department of Urology, Faculty of Medicine, Technische Universität Dresden, Dresden, Germany

2 National Center for Tumor Diseases (NCT), Dresden, Germany: German Cancer Research Center (DKFZ), Heidelberg, Germany; Faculty of Medicine and University Hospital Carl Gustav Carus, Technische Universität Dresden, Dresden, Germany; Helmholtz-Zentrum Dresden - Rossendorf (HZDR), Dresden, Germany

3 National Center for Radiation Research in Oncology (OncoRay), Faculty of Medicine, Technische Universität Dresden, Dresden, Germany

4 Helmholtz-Zentrum Dresden - Rossendorf, Institute of Radiooncology - OncoRay, Dresden, Germany

5 German Cancer Consortium (DKTK), Partner Site Dresden, Dresden and German Cancer Research Center (DKFZ), Heidelberg, Germany

6 Institute of Legal Medicine, Faculty of Medicine, Technische Universität Dresden, Dresden, Germany

# Current address: Center for Regenerative Therapies Dresden (CRTD), Technische Universität Dresden, Dresden, Germany

*** Correspondence to:**

Kati Erdmann

E-Mail: kati.erdmann@uniklinikum-dresden.de

Phone: +49-351-458-15683

ORCID ID: 0000-0003-3717-3637

Supplementary Files

**Table S1:**	**Cell line authentication via STR analysis**
**Figure S1:**	**Validation of DTX resistance in DTXR cells**
**Figure S2:**	**Evaluation of CDDP tolerance – cell growth and apoptosis**

Table S1. Cell line authentication via STR analysis (DU145 cell lines)

STR marker	STR type[1]	Expected in DU145	Purchased control DNA	DU145# CTRL	DU145# RR	DU145Δ CTRL	DU145Δ DTXR
Amelogenin		X, Y	X, Y	X, Y	X, Y	X, Y	X, Y
CSF1PO	simple tetranucleotide repeat	10, 11	10, 11	9, 10, 11	10, 11	10, 11	9, 10, 11, 12
D13S317	simple tetranucleotide repeat	12, 13, 14	12, 13, 14	12, 13, 14	12, 13, 14	12, 13, *	10, 11, 12, 13, *
D16S539	simple tetranucleotide repeat	11, 13	11, 13	11, 12, 13	11, 13	11, 12, 13	11, 12, 13
D18S51	simple tetranucleotide repeat	12, 13	12, *	12, *	11, 12, *	12, *	11, 12, *
D21S11	complex tetranucleotide repeat	30, 33, 34	30, 33, *	30, 32, 33, *	30, 33, *	30, 31, 32, *, *	29, 30, 32, 33, *
D3S1358	compound tetranucleotide repeat	16	16	16	16	16	15, 16
D5S818	simple tetranucleotide repeat	10, 13	10, 13	10, 13	10, 13	10, 13	9, 10, 13
D7S820	simple tetranucleotide repeat	7, 10, 11, 12	7, 10, 11, *	7, 10, 11, *	7, 10, 11, *	7, *, 11, *	7, *, 11, *, 13
D8S1179	simple tetranucleotide repeat	13, 14	13, 14	13, 14	13, 14	13, 14	13, 14, 15
FGA	complex tetranucleotide repeat	22, 23	21, 22, *	22, *	22, *	22, *	21, 22, 23
Penta D	simple penta-rep.	9, 13	9, 13	9, 13	9, 13	9, 13	9, 13
Penta E	simple penta-rep.	12, 14	12, 14	12, 14	11, 12, 14	12, 14	12, 14
THO1	simple tetranucleotide repeat	7	7	7	7	7	7
TPOX	simple tetranucleotide repeat	11	11	11	10, 11	11	11
vWA	compound tetranucleotide repeat	17, 18, 19	17, 18, 19	17, 18, 19	17, 18, *	*, 18, *	17, 18, *
Deviations[2] compared to expected profile:			4	6	7	7	11

(continued on next page)

Continuation of Table S1. Cell line authentication via STR analysis (PC-3 cell lines)

STR marker	STR type[1]	Expected in PC-3	Purchased control DNA	PC-3# CTRL	PC-3# RR	PC-3Δ CTRL	PC-3Δ DTXR
Amelogenin		X	X	X	X	X	X
CSF1PO	simple tetranucleotide repeat	11	11	11	10, 11	11	11
D13S317	simple tetranucleotide repeat	11	11	11	10, 11	11	11
D16S539	simple tetranucleotide repeat	11	11	11	11	11	11
D18S51	simple tetranucleotide repeat	14, 15	14, 15	14, 15	14, 15	14, 15	14, 15
D21S11	complex tetranucleotide repeat	29, 31.2	29, 31.2	29, 31.2	29, 31.2	29, 31.2	29, 31.2
D3S1358	compound tetranucleotide repeat	16	16	16	16	16	16
D5S818	simple tetranucleotide repeat	13	13	13	13	13	13
D7S820	simple tetranucleotide repeat	8, 11	8, 11	8, 11	8, 11	8, 11	8, *
D8S1179	simple tetranucleotide repeat	13	13	13	13	13	13
FGA	complex tetranucleotide repeat	24	24	24	23, 24	24	24
Penta D	simple pentanucleotide repeat	9	9	9	9	9	9
Penta E	simple pentanucleotide repeat	10, 17	10, 17	10, 17	10, 17	10, 17	10, 17
THO1	simple tetranucleotide repeat	6, 7	6, 7	6, 7	6, 7	6, 7	6, 7
TPOX	simple tetranucleotide repeat	8, 9	8, 9	8, 9	8, 9	8, 9	8, 9
vWA	compound tetranucleotide repeat	17	17	17	17	17	17
Deviations[2] compared to expected profile:			0	0	3	0	1

[1] STR types as classified by Urquhart et al. 1994

[2] Deviations compared to expected profile are marked red. * denotes missing alleles. Every STR marker not matching the expected profile is counted as one deviation.

Expected profiles are available on the ATCC website.

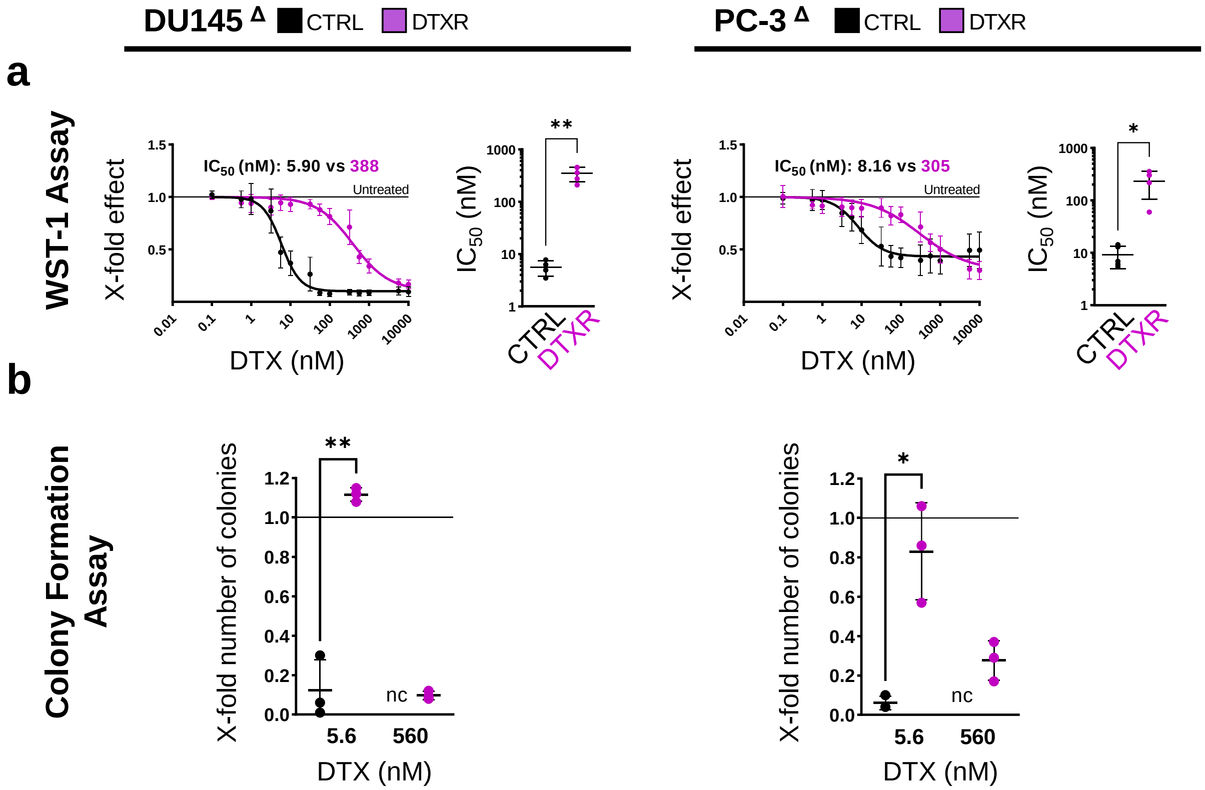

Fig. S1 Validation of DTX resistance in DTXR cells

a: WST-1 assay: Formazan absorbance values of treated cells normalized to untreated cells are plotted and IC_{50} values (noted in the graphs) were calculated from the resulting inhibition curves. Adjacent to the inhibition curves, IC_{50} values of individual experiments are plotted. **b**: Colony formation assay: X-fold numbers of colonies relative to untreated cells are plotted. All data points and error bars in this figure represent Mean$\pm$SD and experiments were repeated for N≥3 times. Non-paired t-tests with Welch's correction were performed to test for statistical significance of the differences between DTXR cells and parental controls. nc: no colonies, *: P<0.05, **: P<0.01.

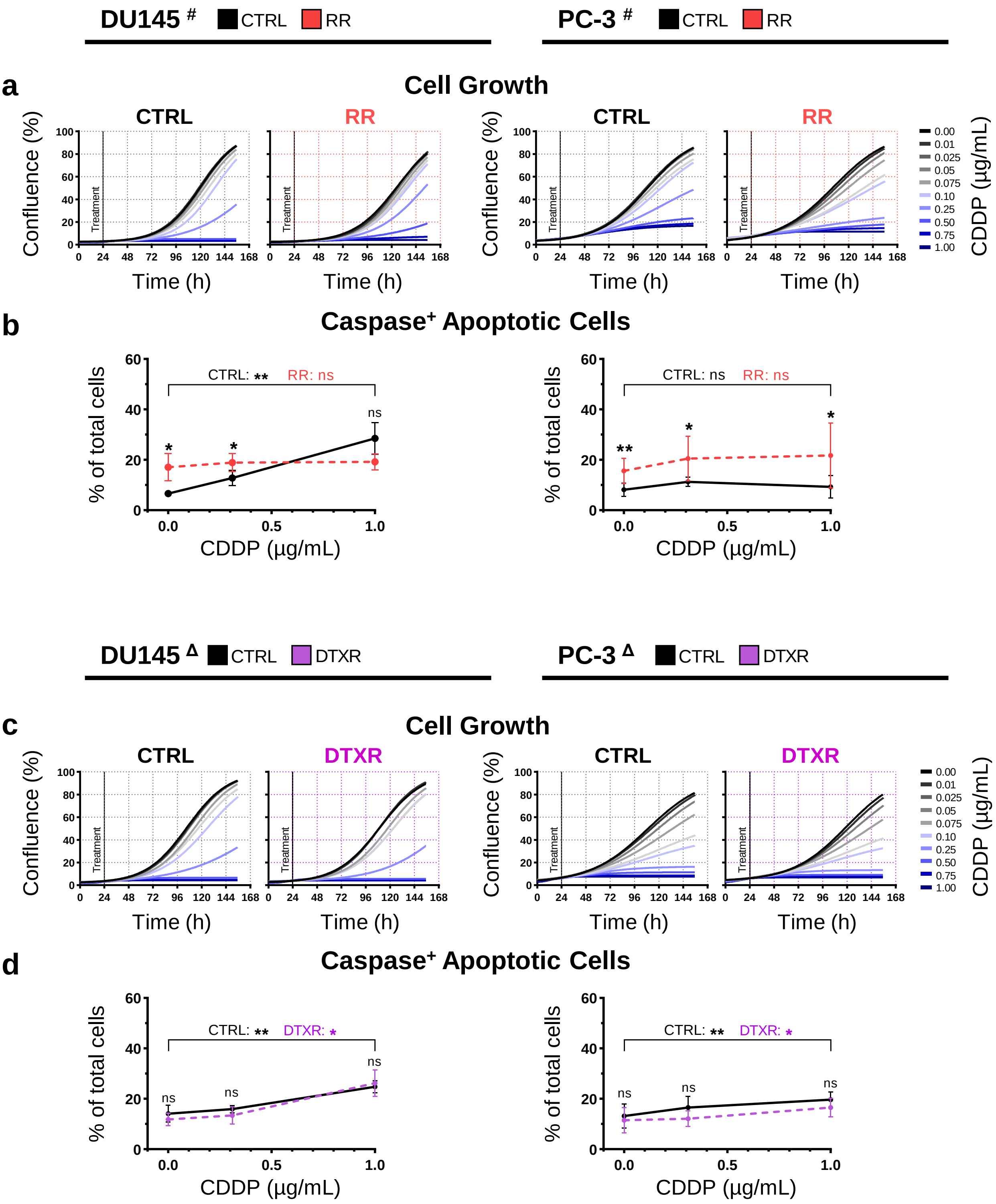

Fig. S2 Evaluation of CDDP tolerance in RR cells, DTXR cells and their parental controls

a and **c**: Cell growth: Cells were tracked in the IncuCyte S3 Live-Cell Analysis System and confluence was determined from acquired images every 6 h. Averaged datapoints from individual experiments (N≥4) are omitted to improve the visibility of fitted curves. **b** and **d**: Measurement of pan-caspase activity: Caspase+ cells are plotted as % of total cells. Data points and error bars represent Mean±SD. All experiments were repeated for N≥3 times. ns: not significant, *: P<0.05, **: P<0.01.
